# Identification of *Thermotoga maritima* MSB8 GH57 α-amylase AmyC as a glycogen-branching enzyme with high hydrolytic activity

**DOI:** 10.1007/s00253-019-09938-1

**Published:** 2019-06-13

**Authors:** Xuewen Zhang, Hans Leemhuis, Štefan Janeček, Mária Martinovičová, Tjaard Pijning, Marc J.E.C. van der Maarel

**Affiliations:** 1 Department of Aquatic Biotechnology and Bioproduct Engineering, Engineering and Technology institute Groningen, University of Groningen, 9747 AG Groningen, Netherlands; 2 Avebe Innovation Center, 9747 AG Groningen, Netherlands; 3 Laboratory of Protein Evolution, Institute of Molecular Biology, Slovak Academy of Sciences, SK-84551 Bratislava, Slovakia; 4 Department of Biology, Faculty of Natural Sciences, University of SS Cyril and Methodius, SK-91701 Trnava, Slovakia; 5 Biomolecular X-ray Crystallography, Groningen Biomolecular Sciences and Biotechnology Institute, University of Groningen, 9747 AG Groningen, Netherlands

**Keywords:** *Thermotoga maritima*, AmyC, Glycogen branching enzyme, Phylogeny, Crystal structure

## Abstract

AmyC, a glycoside hydrolase family 57 (GH57) enzyme of *Thermotoga maritima* MSB8, has previously been identified as an intracellular α-amylase playing a role in either maltodextrin utilization or storage polysaccharide metabolism. However, the α-amylase specificity of AmyC is questionable as extensive phylogenetic analysis of GH57 and tertiary structural comparison suggest that AmyC could actually be a glycogen-branching enzyme (GBE), a key enzyme in the biosynthesis of glycogen. This communication presents phylogenetic and biochemical evidence that AmyC is a GBE with a relatively high hydrolytic (α-amylase) activity (up to 30% of the total activity), creating a branched α-glucan with 8.5% α-1,6-glycosidic bonds. The high hydrolytic activity is explained by the fact that AmyC has a considerably shorter catalytic loop (residues 213–220) not reaching the acceptor side. Secondly, in AmyC, the tryptophan residue (W 246) near the active site has its side chain buried in the protein interior, while the side chain is at the surface in Tk1436 and Tt1467 GBEs. The putative GBEs from three other *Thermotogaceae*, with very high sequence similarities to AmyC, were found to have the same structural elements as AmyC, suggesting that GH57 GBEs with relatively high hydrolytic activity may be widespread in nature.

**Electronic supplementary material:**

The online version of this article (10.1007/s00253-019-09938-1) contains supplementary material, which is available to authorized users.

## Introduction

Glycoside hydrolases (GHs; EC 3.2.1.x) catalyse the hydrolysis of *O*-glycosidic bonds in carbohydrates such as starch. They are ubiquitously present in all kingdoms of life. Well-known GHs are α-amylase (EC 3.2.1.1); the enzyme present in, e.g. saliva and the small intestine, responsible for the degradation of starch; and lactase (E.C. 3.2.1.108), which degrades the milk sugar lactose to glucose and galactose. GHs are classified based on amino acid sequence homology in 152 different families (CAZy) (Cantarel et al. 2009; Lombard et al. 2014). Most GHs have either an inverting or a retaining reaction mechanism as outlined by Koshland (1953).

In essence, GHs catalyse both hydrolysis and transglycosylation reactions, but the ratio varies enormously depending on the type of GH, the substrate concentration and the reaction conditions (Bissaro et al. 2015; Koshland 1953). Typical GHs with almost exclusive hydrolytic activity are isoamylases (EC 3.2.1.68), which hydrolyze the α-1,6-glycosidic linkage in amylopectin (Harada et al. 1972; Li et al. 2013), and α-amylases (EC 3.2.1.1), which hydrolyze the α-1,4-glycosidic linkage in amylose, amylopectin and glycogen (van der Maarel et al. 2002). An example of a GHs with almost exclusive transglycosylating activity are 4-α-glucanotransferases (EC 2.4.1.25) that break an α-1,4-glycosidic linkage in amylopectin or amylose and form a new α-1,4-glycosidic linkage when transferring a part of the donor molecule to the acceptor (Lee et al. 1970; Terada et al. 1999; van der Maarel and Leemhuis 2013).

The glycoside hydrolase family 57 (GH57) was established in 1996 (Henrissat and Bairoch 1996) based on the sequences of two amylolytic enzymes from *Dictyoglomus thermophilum* (Fukusumi et al. 1988) and *Pyrococcus furiosus* (Laderman et al. 1993) that were obviously unrelated to the members of the main α-amylase family GH13 (Janecek et al. 2014). For the family GH57 members, five conserved sequence regions (CSRs) have been established (Zona et al. 2004). Currently, GH57 holds over 2000 protein sequences (CAZy update from February 2019) comprising hydrolytic and transglycosylating enzymes, such as α-amylase, amylopullulanase (EC 3.2.1.41), dual-specificity amylopullulanase/cyclomaltodextrinase (EC 3.2.1.41/54), glycogen-branching enzyme (GBE; EC 2.4.1.18), 4-α-glucanotransferase, and α-galactosidase (EC 3.2.1.22), as well as a non-specified amylase (EC 3.2.1.-) and maltogenic amylase (EC 3.2.1.133) (Blesak and Janecek 2012; Blesak and Janecek 2013; Janecek et al. 2014; Martinovičová and Janeček 2018; Zona et al. 2004). Glycogen-branching enzymes of GH57 play a pivotal role in the synthesis of glycogen, cleaving an α-1,4-glycosidic linkage in the donor substrate subsequently transferring the non-reducing end fragment to the C6 hydroxyl position of an internal glucosyl moiety that acts as the acceptor substrate (α-1,6-transglycosylation).

Ballschmiter et al. (2006) identified AmyC from the thermophilic bacterium *Thermotoga maritima* MSB8 (Taxonomy ID: 243274), an enzyme produced during the exponential growth phase and showing activity towards amylose and soluble starch at high temperature, releasing oligosaccharides. Sequence analysis revealed that AmyC belongs to GH57 and has no signal peptide. Together, the authors concluded that AmyC is an intracellular GH57 α-amylase that may play a role in either maltodextrin utilization or storage polysaccharide metabolism (Ballschmiter et al. 2006).

The crystal structure of AmyC (Dickmanns et al. 2006) showed structural similarity with PDB entry 1UFA, a GH57 enzyme (TT1467) with then unknown function. Santos et al. (2011) determined the crystal structure of another GH57 enzyme, TK1436, from *Thermococcus kodakaraensis* KOD1, and compared its structure with that of AmyC. TK1436 was found to be a GBE; it features a long and flexible so-called catalytic loop (residues 225–245, TK1436 numbering) folding towards the active site with a tyrosine residue at its tip (Tyr233, TK1436 numbering); this loop was shown to be essential for branching activity and proposed to be involved in substrate binding and/or intermediate product stabilization (Palomo et al. 2011; Santos et al. 2011). AmyC showed a considerably shorter catalytic loop, lacking the corresponding tyrosine residue as well as another conserved tryptophan residue lining the active site groove (Trp270, TK1436 numbering). While TK1436 was found to be functional as a tetramer, AmyC is monomeric. The authors proposed that the differences in tertiary and quaternary structure relate to the fact that AmyC only showed hydrolytic activity on starch-like substrates. This hypothesis was further supported by the observation that also TT1467 was characterized as a GBE (PDB entry 3P0B (Palomo et al. 2011)) and features the same structural elements as TK1436, but differs from AmyC regarding those.

Nevertheless, a detailed bioinformatic analysis of GH57 enzymes (Blesak and Janecek 2012) clearly showed that AmyC contains the sequence fingerprint of GBE’s; thus, it remained intriguing why the biochemical characterization of AmyC (Ballschmiter et al. 2006) only revealed hydrolytic and not transglycosylation (branching) activity. We therefore investigated the phylogeny, activity and three-dimensional structure of AmyC in more detail. This communication presents biochemical evidence in support of the in silico analysis that AmyC is indeed a GBE with relatively high hydrolytic activity (up to 30% of the total activity), and suggests which structural features are responsible for its specificity. Finally, three putative GH57 GBEs are identified based on structural homology to AmyC, suggesting that GH57 GBEs with relatively high hydrolytic activity are more widespread in mesophilic and thermophilic microorganisms.

## Materials and methods

### Materials

Amylose V was provided by Avebe (Veendam, Netherlands). Lithium bromide was obtained from Acros Organics. Isoamylase (specific activity 260 U/mg), pullulanase M1 (EC 3.2.1.41, specific activity 34 U/mg) and β-amylase (EC 3.2.1.2, specific activity 10,000 U/mL) were purchased from Megazyme (Wicklow, Ireland). All other chemicals were from Sigma-Aldrich (Zwijndrecht, Netherlands)

### Sequence and evolutionary comparison

All full-length protein sequences (Supplementary Table S1) were retrieved from the UniProt knowledge database (http://www.uniprot.org/) (Apweiler 2014) and/or from GenBank (https://www.ncbi.nlm.nih.gov/genbank/) (Benson et al. 2014). The alignment was done for AmyC from *T. maritima* and the three characterized GBEs from *T. kodakaraensis*, *T. thermophilus* and *P. horikoshi*, for which also their three-dimensional structures have been determined—using the program Clustal-Omega with default parameters (http://www.ebi.ac.uk/Tools/msa/clustalo/) (Sievers et al. 2011).

For all 64 GH57 enzymes and proteins (Supplementary Table S1), their five well-established conserved sequence regions (CSRs) (Zona et al. 2004) were identified according to previous bioinformatics analyses (Blesak and Janecek 2012, 2013; Janecek and Blesak 2011; Martinovičová and Janeček 2018). The evolutionary tree was calculated based on the alignment of five CSRs mentioned above as a Phylip-tree type using the neighbour-joining clustering (Saitou and Nei 1987) and the bootstrapping procedure (Felsenstein 1985) (the number of bootstrap trials used was 1000) implemented in the Clustal-X package (Larkin et al. 2007). The tree was displayed with the program iTOL (http://itol.embl.de/) (Letunic and Bork 2006). Sequence logos were created using the WebLogo 3.0 server (http://weblogo.threeplusone.com/) (Crooks et al. 2004) for CSRs of all 40 GBE sequences as well as of the single AmyC from *T. maritima*.

### Expression and purification of AmyC

A codon-optimized gene (Genbank ID: MK704497) encoding the GBE from *T. maritima* SMB8 (AmyC) was synthesized by Baseclear (Leiden, Netherlands), and cloned into pRSET B (Invitrogen) behind the His-tag sequence of the vector. Gene sequence details are provided in the supplemental information. AmyC was overexpressed in *Escherichia coli* BL21(DE3), cultivated in Luria-Bertani (LB) medium (10 g/L of tryptone, 5 g/L yeast extract and 10 g/L NaCl) supplemented with 100 μg/mL ampicillin. GBE expression was induced with 0.1 mM IPTG when the culture had an OD_600_ of 0.8; the induction was carried out at 16 °C for 20 h. Cells were harvested by centrifugation (5000×*g*, 10 min, 4 °C), washed twice with 10 mM sodium phosphate buffer pH 7.0, and lysed using a high-pressure homogenizer (Emulsiflex-B15; Avestin, Canada) in two cycles at 9.0 MPa and room temperature. A cell-free extract was obtained after centrifugation (20,000×*g*, 30 min, 4 °C). AmyC was purified in two steps: first, the cell-free extract was subjected to 70 °C for 15 min, followed by removal of the denatured proteins by centrifugation (20,000×*g*, 30 min, 4 °C). The His-tagged AmyC was purified using the HisPurTM Ni-NTA Resin (Thermo Fisher Scientific, Waltham, USA) according to the manufacturer’s protocol. Protein concentration was quantified using the Quick Start™ Bradford Protein Assay kit (Bio-Rad Laboratories, Hercules, USA). The purity and molecular mass of the proteins were checked by SDS-PAGE.

### Enzyme activity assays

The enzyme activity was analysed using the iodine staining assay and monitoring the decrease of absorbance of the glucan-iodine complex (Boyer and Preiss 1978). Amylose V with an average DP of 700 was selected as substrate because it has no detectable α-1,6-linkages by NMR; so, any α-1,6-linkage detected is the primed produced by the result of the action of the GBE.

Amylose V (0.125% (*w*/*v*) dissolved in 50 mM sodium phosphate buffer (pH 7.0) was incubated with 132.5 μg/mL AmyC at 50 °C. Ten microliters of aliquot was taken into 96-well plate, and 150 μL iodine reagent (aqueous solution of 0.0127% I_2_ and 0.035% KI) was added, and the absorption at 660 nm was determined. One unit of enzyme activity is defined as the amount of enzyme that gives a decrease in absorbance of the amylose/iodine complex of 1.0 absorbance unit per minute at 660 nm (Palomo et al. 2011).

The influence of Ca^2+^ on AmyC activity was tested at 50 °C to 80 °C in 50 mM Tris-Cl buffer, and the pH 7.5 was adjusted at a series reaction temperatures. 0.1, 1, 3 and 5 mM CaCl_2_ were applied in the reaction. The activity was measured by iodine assay described as above.

The hydrolytic and transglycosylation activity of AmyC with amylose V as substrate were determined by measuring the increase in reducing ends by the bicinchoninic acid (BCA) method before and after debranching the product, respectively. Amylose V was dissolved in 1 M sodium hydroxide and then neutralized to pH 7.0. A mixture of 0.125% (*w*/*v*) amylose V in 50 mM sodium phosphate buffer (pH 7.0) and 132.5 μg/mL AmyC was incubated at 50 °C. Samples of 500 μL were taken at different time points and the AmyC was inactivated by incubating the samples at 100 °C for 10 min. To debranch the product, 200 μL sample was mixed with 1 μL 1 M citrate acid, 1 U isoamylase, 0.7 U pullulanase and 1 μL 1 M CaCl_2_ and then incubated at 40 °C for 16 h. The hydrolytic activity was measured by following the increase in reducing ends during the reaction as each product of hydrolysis bears a terminal, reducing glucose residue. Transglycosylation, or branching activity, was measured by treating the reaction product with the debranching enzymes isoamylase and pullulanase, enzymes that specifically hydrolyze α-1,6-linkages; the product of the specific hydrolysis of α-1,6-linkage will also bear a terminal, reducing glucose residue. The increase in reducing ends is the amount of reducing ends after debranching minus the amount of reducing ends before debranching, a direct result of the transglycosylation/branching activity. One unit branching activity is defined as 1 μmol of α-1,6-linkage synthesized per minute and one unit hydrolytic activity is defined as 1 μmol of reducing end synthesized per minute.

### Influence of pH and temperature on activity

The influence of pH on AmyC activity was measured at 50 °C in 50 mM sodium phosphate buffer (pH 6.0 to 9.0) by using the iodine assay as described above. The influence of temperature on AmyC activity was determined at pH 7.0 in 50 mM sodium phosphate buffer using the activity assay mixture incubated at temperature ranging 40 to 90 °C.

### High performance anion exchange chromatography

Oligosaccharide analysis was carried out by high-performance anion exchange chromatography (HPAEC) on a Dionex ICS-3000 system (Thermo Scientific) equipped with a 4 × 250 mm CarboPac PA-1 column. A pulsed amperometric detector with a gold electrode and an Ag/AgCl pH reference electrode were used. The system was run with a gradient of 30–600 mM NaAc in 100 mM NaOH 1 mL/min. Chromatograms were analysed using Chromeleon 6.8 chromatography data system software (Thermo Scientific). A mixture of glucose, maltose, maltotriose, maltotetraose, maltopentaose, maltohexaose and maltoheptaose was used as reference. AmyC-modified product was dialyzed using dialysis tubing with a cutoff size of 100 Da to 500 Da in ultrapure water. Two milligrams of dry material was dissolved into 1 mL 5 mM sodium acetate buffer pH 5.0 with 5 mM CaCl_2_. Five hundred microliters of solution was mixed with 2.5 U isoamylase and 1.75 U pullulanase, and incubated at 40 °C for 16 h.

### ^1^H-NMR spectroscopy

^1^H-NMR spectra were recorded at a probe temperature of 323 K on a Varian Inova 500 spectrometer (NMR Center, University of Groningen). Before NMR analysis, samples were exchanged twice in D_2_O (99.9% D atom, Sigma-Aldrich) with intermediate lyophilization, and then dissolved in 0.6 mL D_2_O. Spectra were processed using MestReNova 5.3 software (Mestrelabs Research SL, Santiago de Compostella, Spain), using Whittaker Smoother baseline correction and zero filling to 32 k complex points. The degree of branching is calculated as follows:

$$ \mathrm{Degree}\ \mathrm{of}\ \mathrm{branching}=\frac{S_{\upalpha -1,6}}{S_{\upalpha -1,4}+{S}_{\upalpha -1,6}} $$

S_α-1,6_ is the peak area of α-1,6, integrated from NMR spectra; S_α-1,4_ is the peak area of α-1,4, integrated from NMR spectra.

### Structural homology modelling

The crystal structures of AmyC (PDB entry 2B5D), *T. kodakaraensis* TK1436 GBE (PDB entry 3N98; (Santos et al. 2011)), *T. thermophilus* TT1467 GBE (PDB entry 3P0B; (Palomo et al. 2011)) and *T. litoralis* 4-α-glucanotransferase in complex with acarbose (PDB entry 1K1Y; (Imamura et al. 2003)) were superimposed. Homology models of *Mesotoga prima*, *Kosmotoga olearia* and *Kosmotoga pacifica* putative GBEs were generated using the Phyre server in intensive mode (Kelley et al. 2015). Structural figures were prepared using PyMOL (The PyMOL Molecular Graphics System, Version 2.0 Schrödinger, LLC).

## Results

### Sequence analysis of GH57 GBEs

Analysis of the GH57 GBE sequences and phylogenetic tree construction was performed as described in the ‘Materials and methods’ section. Sequences of 40 GBEs (Supplementary Table S1) were collected based on the recent exhaustive in silico analysis of the entire α-amylase family GH57 (Martinovičová and Janeček 2018) that, of all 1602 GH57 sequences taken from the CAZy database (Cantarel et al. 2009), yielded 546 GBEs. Forty GBEs were selected in an effort to obtain a representative sample of GBE sequences having, in addition to AmyC from *T. maritima* (Ballschmiter et al. 2006; Dickmanns et al. 2006), the biochemically characterized enzymes from *T. kodakaraensis* (Murakami et al. 2006; Santos et al. 2011), *T. thermophilus* (Palomo et al. 2011) and *P. horikoshi* (Na et al. 2017), accompanied by a range of hypothetical GBEs covering various taxa from both *Bacteria* and *Archaea* including all available sequences from the phylum *Thermotogae*. In order to perform as relevant as possible analysis and in accordance with previous in silico studies (Blesak and Janecek 2012; Blesak and Janecek 2013; Janecek and Blesak 2011; Martinovičová and Janeček 2018), the set of sequences was completed by 23 biochemically characterized family GH57 members representing other enzyme specificities, accompanied by one putative representative of the α-amylase-like protein (Supplementary Table S1).

**Fig. 1:**
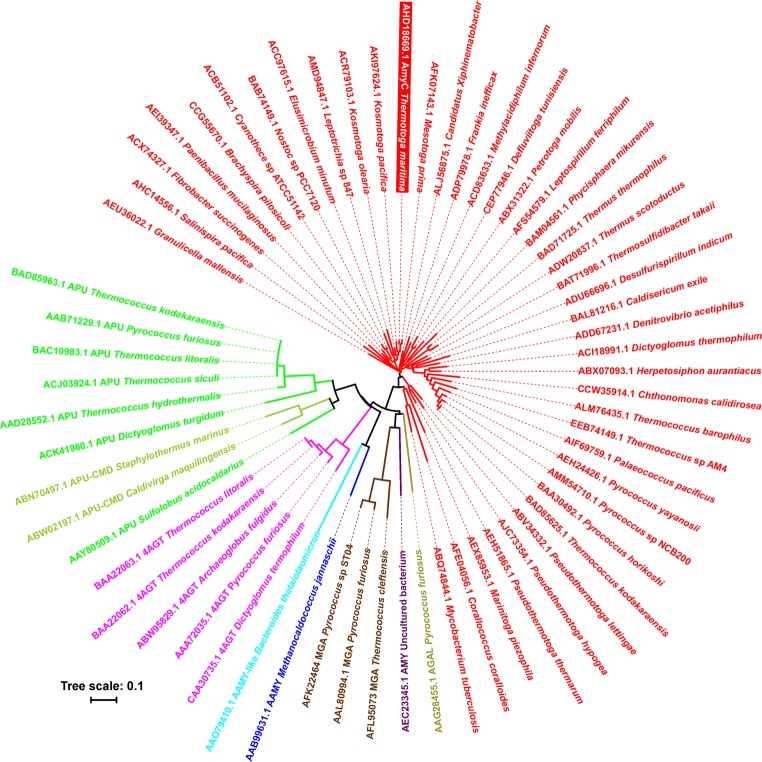
Evolutionary tree of all 64 GH57 enzymes and proteins analysed in the present study. The tree is based on the alignment of five CSR characteristics of family GH57. The individual enzyme specificities are distinguished from each other by different colours (for details, see Supplementary Table S1). The label of each GBE (shown in red) consists of the GenBank accession number, letter “A” or “B” indicating the archaeal and bacterial origin, respectively, and the name of the organism; for remaining enzyme specificities, also the abbreviation of the enzyme name is added as follows: AAMY, α-amylase; AAMY-like protein, α-amylase-like protein; 4AGT, 4-α-glucanotransferase; APU, amylopullulanase; APU-CMD, amylopullulanase/cyclomaltodextrinase; AMY, non-specified amylase; MGA, maltogenic amylase; AGAL, α-galactosidase. The four GBEs with known three-dimensional structures are marked by an asterisk. The AmyC from *Thermotoga maritima* is emphasized by colour inversion. All GBEs from the phylum *Thermotogae* (i.e. genera *Thermotoga*, *Kosmotoga*, *Mesotoga*, *Defluviitoga*, *Petrotoga* and *Pseudothermotoga*) are signified by red full circles

**Fig. 2:**
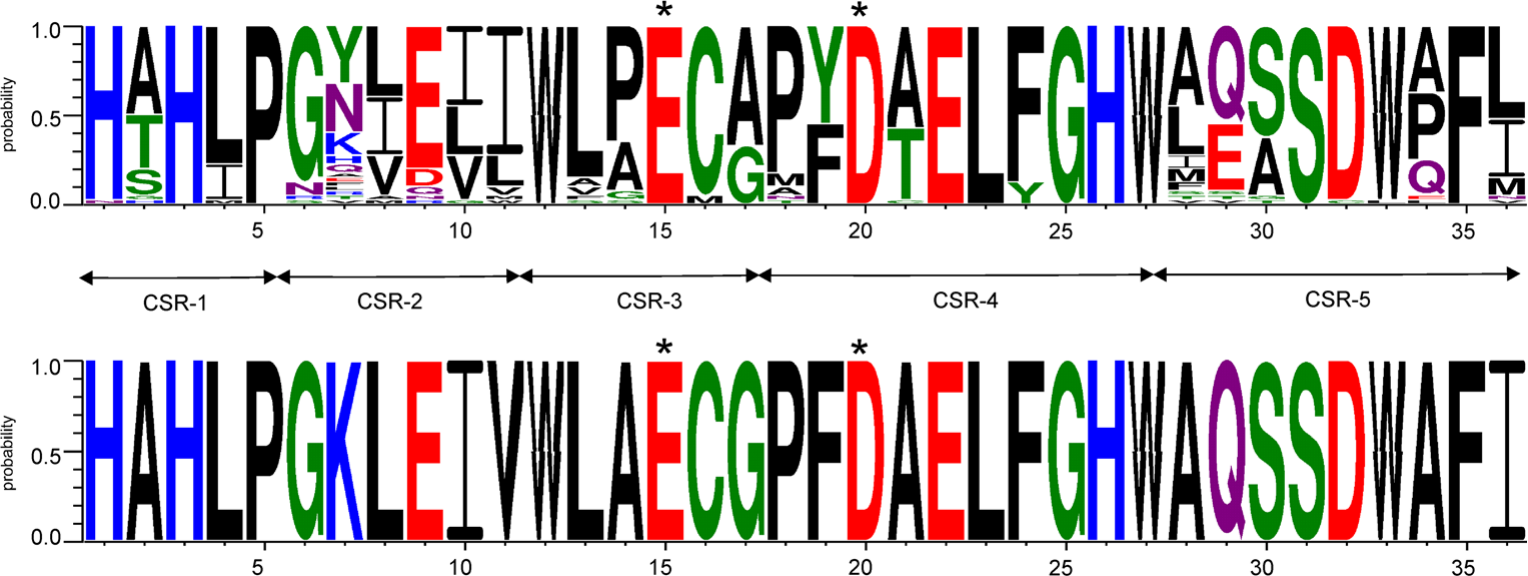
Sequence logos of 40 GBEs from the family GH57 analysed in the present study (top) and of the AmyC from *Thermotoga martitima* (bottom). CSR-1, residues 1–5; CSR-2, residues 6–11; CSR-3, residues 12–17; CSR-4, residues 18–27; CSR-5, residues 28–36. The catalytic nucleophile (at position 15, glutamate) and the proton donor (at position 20, aspartate) in both logos are indicated by asterisks

The evolutionary tree constructed of these selected sequences shows that the (putative) GH57 GBEs cluster together (Fig. 1). Comparison of the CSRs of the 40 (putative) GH57 GBEs reveals that most, but not all, of the amino acid residues of the CSRs are conserved (Fig. 2). AmyC was also alignment with the three characterized GH57 GBE, demonstrating their similarity, though at the same time also revealing that some loops are distinct (Fig. S1).

### Activity

**Fig. 3:**
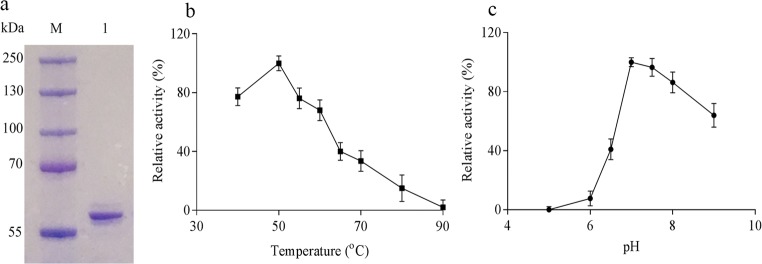
SDS-PAGE of purified recombinant AmyC (**a**), and effect of temperature (**b**) and pH (**c**) on AmyC activity. The activity was measured by iodine staining assay. Amylose V was used as substrate with DP 700, and the samples were measured every 30 min and total incubation time is 3 h

AmyC was over expressed in *E. coli* and purified to homogeneity as judged by SDS-page (Fig. 3a). The previous studies report optimal conditions of 90 °C and pH 8.5 (Ballschmiter et al. 2006; Dickmanns et al. 2006). In a first approach, the activity of purified recombinant enzyme was tested at 90 °C and pH 8.5 by using the iodine staining assay and amylose V as substrate. The absorbance of glucan-iodine complex did not change, which showed that the recombinant AmyC was not active at these conditions. Subsequently, the influence of temperature and pH on AmyC activity was investigated in detail. AmyC showed activity at temperatures of 80 °C and below. Maximum activity was found at 50 °C and pH 7.0 (Fig. 3). AmyC was not active in the presence of Ca^2+^ at high temperature, which is in agreement with Ballschmiter et al. (2006).

**Fig. 4:**
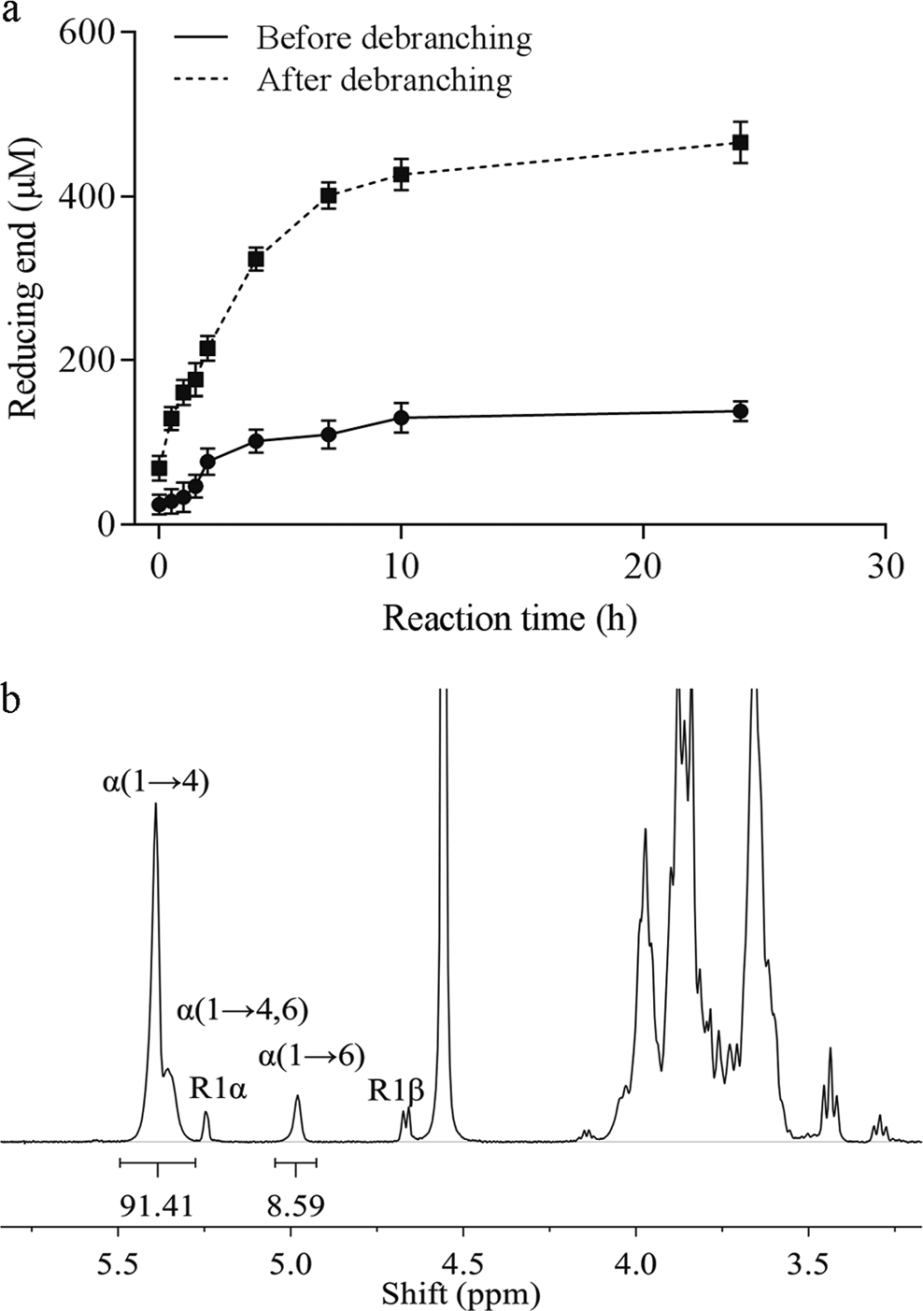
The branching and hydrolytic activity of AmyC (**a**), and ^1^H-NMR spectra of AmyC branched α-glucan (**b**). The activities are quantified by following the increase in reducing ends over time. Amylose V (0.125%) was incubated with AmyC (132.5 μg/mL) in phosphate buffer with pH 7.0 at 50 °C. The samples were debranched by isoamylase and pullulanase. ^1^H-NMR spectra of branched α-glucan made by AmyC from amylose V in D_2_O, recorded at 50 °C

AmyC was incubated with amylose V and samples were taken in time. The amount of reducing ends increased gradually during the reaction (Fig. 4a), due to the α-amylase activity of AmyC. However, importantly, also a clear increase in reducing ends was found when the product of the incubation of amylose V and AmyC was treated with isoamylase/pullulanase (Fig. 4a). From the increase in reducing ends before and after debranching, the hydrolytic and transglycosylating activity were calculated. The total activity of AmyC is 12 mU/mg protein calculated from reducing end at 0 h and 2 h, consisting of a transglycosylating activity of 9 mU/mg protein and a hydrolytic activity of 3 mU/mg protein.

### ^1^H-NMR spectroscopy and chain length distribution

To confirm the branching activity and determine the type of glycosidic linkages introduced by AmyC, the reaction product was submitted to ^1^H-NMR (Fig. 4b). The ^1^H-NMR spectrum of AmyC-modified amylose V showed a clear α-1,6 signal at δ4.98, originating from the H1 in the residue 1,4-α-glucose-1,6. An α-1,4 signal was found at δ5.36, originating from the H1 in the residues 1,4-α-glucose-1,4 and 1 → 4,6-α-glucose-1,4. These two peak areas gave the degree of branching of 8.5%, which is a bit lower than TtGBE of 9.2% and TkGBE of 9.4% (data not shown). The ^1^H-NMR spectrum also shows clear reducing end signals at δ5.25 and δ4.66, being respectively the α- and the β-anomer.

**Fig. 5:**
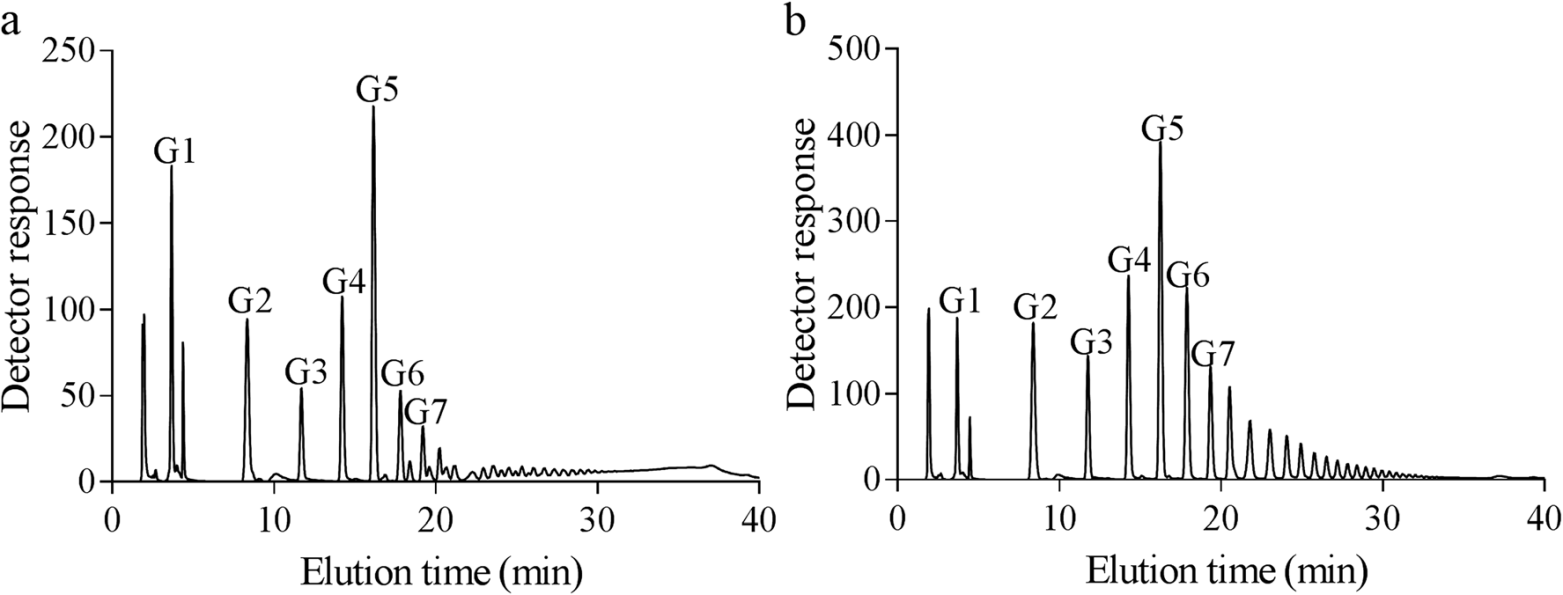
HPAEC analysis of the branched α-glucan derived from amylose V by the action of AmyC. **a** The final branched product; **b** debranched final product by isoamylase and pullulanase

**Fig. 6:**
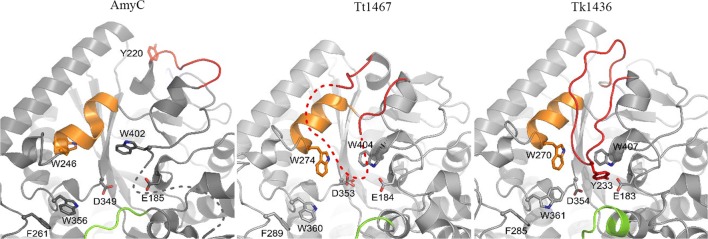
Structural comparison of GH57 GBEs, showing AmyC (left), TT1467 (middle) and TK1436 (right) with structural differences around the active site groove; highlighted are the catalytic loop (red), the helix with the gatekeeper tryptophan residue (orange), lid 2 (green) and some of the other aromatic residues as well as the two catalytic residues. Importantly, in AmyC, the catalytic loop is too short to reach the active site as it does in TT1467 and TK1436; e.g. tyrosine 220 in this loop is at about 30-Å distance from the catalytic site. Moreover, residue W246 of AmyC is in a buried conformation, unable to function as a binding platform for acceptor carbohydrates. Residues not visible in the structures are indicated with a dotted line

The 24-h product derived from amylose V was analysed by HPAEC-PAD before and after debranching (Fig. 6). AmyC produced mainly branched products and linear oligosaccharides of DP 1 to 8 as by-products with minor amounts of short-chain-branched oligosaccharides, visible as small peaks directly next to the larger linear oligosaccharide peaks (Fig. 5a). After debranching, more short-chain linear oligosaccharides and in addition longer linear oligosaccharides were found, representing the newly synthesized side chains (Fig. 5b). AmyC introduces side chains ranging from DP 2 up to DP 30, with DP 5 as the most abundant side chains (Fig. 5b). The average chain length is 6.6.

### Structural analysis of AmyC

**Fig. 7:**
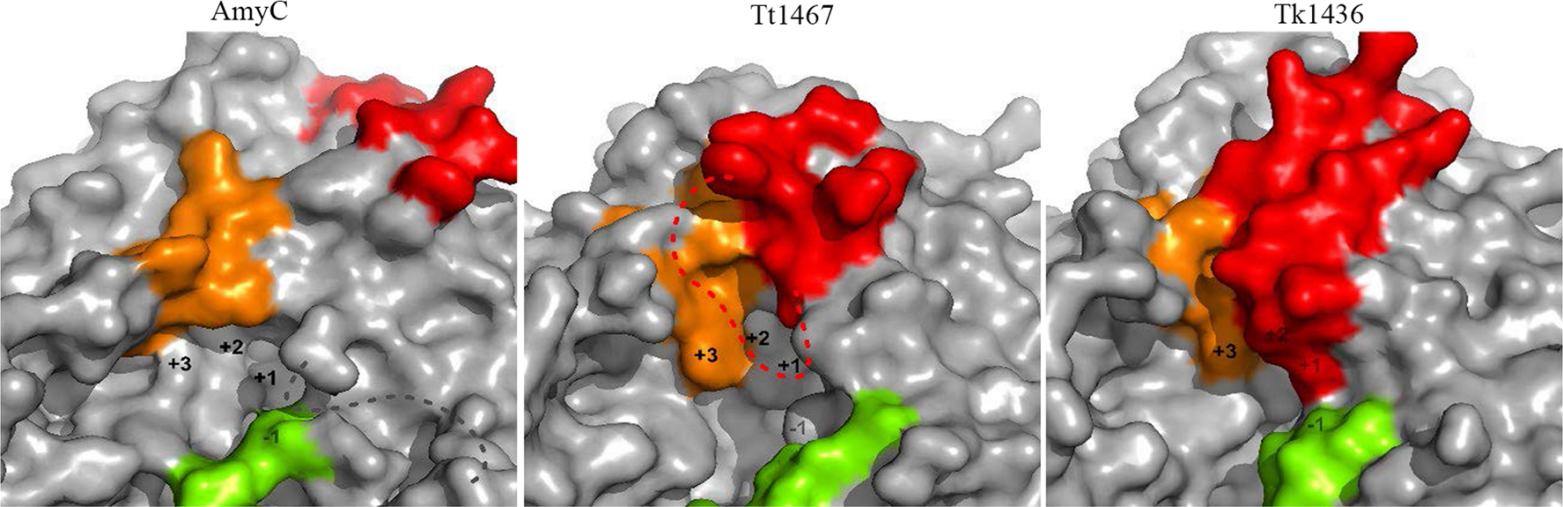
Surface representations of GH57 GBEs, showing AmyC (left), TT1467 (middle) and TK1436 (right). Catalytic loop (red); distorted helix (orange); lid 2 (green). The approximate positions of one donor subsite (−1) and three acceptor subsites (+1 to +3) are derived from a superposition with the structure of *T. litoralis* 4-α-glucanotransferase (TlGT) complexed with acarbose (not shown). As a result of the much shorter catalytic loop (red) in AmyC, its active site groove is less occluded at acceptor subsites

Superposition of AmyC (PDB entry 2B5D) with GH57 GBEs TT1467 (PDB entry 3P0B) and TK1436 (PDB entry 3N98) resulted in root mean square deviations of 1.21 and 1.34 Å (on Cα atoms), respectively, in accordance with their very similar core architecture (Fig. 6). Differences are mainly observed in a few loops, some of which are near the proposed substrate-binding groove. Importantly, the loop of AmyC (residues 213–220) equivalent to the catalytic loop of TT1467/TK1436 is 12 or 11 residues shorter, and, as noted by Santos et al. (2011), cannot reach the acceptor subsites of the active site groove; residue Tyr220 in this loop lies at the side of domain A, about 30 Å from the catalytic site. A second distinct feature of AmyC is the helical element comprising residues 239–246; it is shifted towards the catalytic site and has an imperfect α-helical conformation. This α-helix carries Trp246, equivalent to the gatekeeper Trp274/Trp270 of TT1467/TK1436, but with a side-chain conformation that buries it in the protein interior. Third, the loop connecting the 2nd and 3rd long α-helix in domain C, designated ‘lid 2’ in *T. litoralis* 4-α-glucanotransferase (TlGT), is only partly conserved and has a conformation that brings it closer to the active site groove. Finally, at the lower end of the active site groove, the loop connecting helix α1 and strand β1, carrying residue F24, runs different from other GH57 GBEs (not shown). From Table 1, it is obvious that of the aromatic ‘gatekeeper’ residues of TT1467/TK1436, residues Trp29, Trp402 and Trp411 of AmyC are conserved; however, Trp246 cannot function due to its buried conformation. Moreover, two of the five other aromatic residues contributing to a hydrophobic substrate-binding groove are at a different position or absent in AmyC. Taken together, the AmyC active site groove, compared to TT1467/TK1436, is more open at the acceptor-binding end (Fig. 7) and has likely less affinity for acceptor carbohydrates.

**Table 1:** Comparison of important residues in GH57 GBEs

	*T. kodakaraensis* TK1436	*T. thermophilus* TT1467	*T. maritima* AmyC	*K. olearia*
				*K. pacifica*
				*M. prima*
Nucleophile	E183	E184	E185	Conserved
Acid/base	D354	D353	D349	Conserved
Polarizer of acid/base	H10	H9	H10	Conserved
Aromatic gatekeepers	W28	–	W29	Conserved
	W270	W274	– (W246 buried)	As in AmyC
	W407	W404	W402	Conserved
	W416	W413	W411 not visible	W present but position difficult to predict
Other aromatics	W22	W21	–	–
	F24	F23 (double conform.)	– (F24 at different position)	– (As in AmyC)
	F285	F289	F261	Conserved
	W361	W360	W356	Conserved
	F470	F461	F466 (no side chain modelled)	Conserved
Other near active site	H12	H11	H12	Conserved
	S466	S462	S462	Conserved
	D467	D463	D463	Conserved
	R261	R265	R237	Conserved
Catalytic loop	226–245 (20 res.)	227–248 (22 res.;	214–220 (7 res.)	Short as in AmyC
		235–242 not visible		
	Y233 (tip)	Y236 ? (not visible)	No equivalent	No equivalent
Lid 2	471–476 LITTGQ	464–470 LMETGQ	467–472 IMTTRT closer to active site	FIMTTxT/FIITTxT

### Structural homology modelling of putative GH57 branching enzymes

Structural homology models of the putative GH57 GBEs from *Mesotoga prima*, *Kosmotoga olearia* and *Kosmotoga pacifica* were generated using the Phyre server (Kelley et al. 2015). The generated homology models of the *M. prima*, *K. olearia* and *K. pacifica* putative GBEs were superimposed with the AmyC crystal structure, resulting in very low root mean square deviations of 0.22, 0.23 and 0.17 Å, for 452, 415 and 434 Cα atoms, respectively.

## Discussion

AmyC from *T. maritima* (Dickmanns et al. 2006) was originally described as a family GH57 α-amylase randomly hydrolyzing amylose and soluble starch forming glucose, maltose and maltotriose as the main products (Ballschmiter et al. 2006). However, the eventual α-glucan branching activity was first ascribed to AmyC based on a detailed in silico sequence analysis (Blesak and Janecek 2012) mainly due to presence of a cysteine residue (Cys186) in the CSR-3 (Fig. 2), which was suggested to be a clear branching enzyme sequence feature. Additional support for AmyC branching activity is the presence of a tyrosine (Tyr220) corresponding with Tyr236 of *T. thermophilus* GBE (Fig. S1). This residue is positioned in a loop (235_PYGEAALG) between the CSR-3 and CSR-4; this loop (also called the catalytic loop) was considered essential for enzyme specificity because the Y236A mutant lost the branching activity with simultaneous acquiring of an increased hydrolytic ability (Palomo et al. 2011). Although the sequence alignment (Fig. S1) indicates a 12-residue deletion in the AmyC a few residues after the functionally important tyrosine, its presence in AmyC seems to be conserved. It is worth mentioning that this residue is not conserved invariantly in all GBEs of the present study (data not shown); this fact was observed previously when analysing more than 150 hypothetical GBE sequences (Blesak and Janecek 2012).

Despite the three short regions between the CSR-3 and CSR-4, where AmyC possesses two deletions and one insertion in comparison with the three confirmed GBEs, AmyC unambiguously exhibits with them a high, i.e. more than 47%, sequence similarity (Fig. S1). Sharing the unambiguous GBE sequence features is even more convincing within the family GH57 CSRs (Fig. 2). The AmyC sequence logo does have all the features identified as ‘sequence fingerprints’ of GBE specificity (Blesak and Janecek 2012): (i) a cysteine in the position 16, (ii) a leucine in the position 23 and (iii) a phenylalanine succeeded by a hydrophobic non-aromatic residue in positions 35 and 36, respectively (Fig. 2).

Re-classification of AmyC from *T. maritima* as a family GH57 GBE is further supported by its position in the evolutionary tree (Fig. 1). Within the tree, the hypothetical GBE representatives originating from the phylum *Thermotogae* have been found in three different parts: (i) genera *Thermotoga*, *Mesotoga* and *Kosmotoga* (families *Thermotogaceae* and *Kosmotogaceae*); (ii) *Pseudothermotoga* (family *Thermotogaceae*); and (iii) *Defluviitoga* and *Petrotoga* (family *Petrotogaceae*). In any case, the assignment of the AmyC from *T. maritima* to GBEs is self-evident because the representatives of all remaining family GH57 enzyme specificities, such as α-amylase, amylopullulanase and 4-α-glucanotransferase (Supplementary Table S1), form their own branches and/or clusters clearly separated from all GBEs (Fig. 1).

The biochemical characteristics support the conclusion from the phylogenetic and sequence analyses that AmyC is a functional branching enzyme converting amylose into a branched α-glucan with 8.5% branches. The ^1^H-NMR spectrum shows clear reducing end signals at δ5.25 and δ4.66, being respectively the α- and the β-anomer, representing the hydrolysis products. The hydrolytic, i.e. α-amylase activity found in this study (3 mU/mg), is considerably lower than the activity reported by Ballschmiter et al. (2006). In the present study, the BCA method was used to measure the amount of reducing ends, while Ballschmiter et al. (2006) used the DNS method, which is known to give erroneously high estimates of glycoside hydrolase activity (Gusakov et al. 2011; McCleary and McGeough 2015), including α-amylase (Robyt and Whelan 1972). McCleary and McGeough (Robyt and Whelan 1972) concluded that DNS method should only be used to qualitatively measure glycoside hydrolase activity. The activities reported by Ballschmiter et al. (Dickmanns et al. 2006) therefore should be treated cautiously and should not be compared to activities found with other methods to measure reducing ends.

The homology models of the *M. prima*, *K. olearia* and *K. pacifica* putative GBEs have an architecture highly similar to that of AmyC (Fig. S2). Only the loop connecting domains B and C was modelled differently in the three homologues, likely due to the fact that in the AmyC structure, this loop is not visible. The comparison in Table 1 shows that virtually all the specific features of AmyC described above are conserved, including the shortened catalytic loop, the shifted helical element and lid 2. Therefore, it is very likely that, like AmyC, the *M. prima*, *K. olearia* and *K. pacifica* GBEs have a relatively high hydrolytic activity among GH57 branching enzymes. The identification of putative GBEs in the mesophilic and thermophilic bacteria *M. prima*, *K. olearia* and *K. pacifica* suggests that AmyC is not unique, but that GH57 GBEs with relatively high hydrolytic activity are widespread in such organisms.

## Electronic supplementary material

ESM 1 (PDF 531 kb)
