# Supplementary material for: Identification of Thermotoga maritima MSB8 GH57 α-amylase AmyC as a glycogen-branching enzyme with high hydrolytic activity
Source: Appl Microbiol Biotechnol. 2019 Jun 13;103(15):6141–51. doi: 10.1007/s00253-019-09938-1 (PMC6616209; doi:10.1007/s00253-019-09938-1)
Supplement: Supplementary file 1 — (PDF 531 kb) [file 253_2019_9938_MOESM1_ESM.pdf]

Paper title:

Identification of *Thermotoga maritima* MSB8 GH57 α-amylase AmyC as a glycogen branching enzyme with high hydrolytic activity

Journal: Applied Microbiology and Biotechnology

Authors:

Xuewen Zhang[1], Hans Leemhuis[1,2], Štefan Janeček[3,4], Mária Martinovičová[4], Tjaard Pijning[5], and Marc J.E.C. van der Maarel[1]*

Affiliations:

[1]Department of Aquatic Biotechnology and Bioproduct Engineering, Engineering and Technology Institute Groningen, University of Groningen, Groningen 9747 AG, Netherlands

[2]Avebe Innovation Center, Groningen 9747 AA, Netherlands

[3]Laboratory of Protein Evolution, Institute of Molecular Biology, Slovak Academy of Sciences, SK-84551 Bratislava, Slovakia

[4]Department of Biology, Faculty of Natural Sciences, University of SS Cyril and Methodius, SK-91701 Trnava, Slovakia

[5]Biomolecular X-ray Crystallography, Groningen Biomolecular Sciences and Biotechnology Institute, University of Groningen, Groningen 9747 AG, Netherlands

*Corresponding author; E-mail: m.j.e.c.van.der.maarel@rug.nl

Table S1. List of GH57 sequences used in the present *in silico* analysis.

No.	Enzyme[a]	Domain	Source[b]	GenBank	UniProt	Length	CSR1-CSR5
1	GBE	Bacteria	**Thermotoga maritima* – AmyC	AHD18669.1	UPI00006C21D0	528	10–467
2	GBE	Bacteria	**Thermus thermophilus*	BAD71725.1	Q5SH28	520	9–464
3	GBE	Archaea	**Pyrococcus horikoshii*	BAA30492.1	O50094	560	10–472
4	GBE	Archaea	**Thermococcus kodakarensis*	BAD85625.1	Q5JDJ7	675	10–471
5	GBE	Bacteria	*Brachyspira pilosicoli*	CCG55670.1	K0JH67	529	11–472
6	GBE	Bacteria	*Caldisericum exile*	BAL81216.1	I0GKC9	530	11–470
7	GBE	Bacteria	*Candidatus Xiphinematobacter*	ALJ56875.1	A0A0P0GIN7	527	11–470
8	GBE	Bacteria	*Chthonomonas calidirosea*	CCW35914.1	S0EVS6	581	15–506
9	GBE	Bacteria	*Corallococcus coralloides*	AFE04056.1	H8MV84	994	10–464
10	GBE	Bacteria	*Cyanothece* sp. ATCC 51142	ACB51102.1	B1WYT5	544	15–474
11	GBE	Bacteria	*Defluviitoga tunisiensis*	CEP77946.1	A0A0C7NJ35	535	13–475
12	GBE	Bacteria	*Denitrovibrio acetiphilus*	ADD67231.1	D4H3E7	525	11–468
13	GBE	Bacteria	*Desulfurispirillum indicum*	ADU66696.1	E6W2E6	533	15–474
14	GBE	Bacteria	*Dictyoglomus thermophilum*	ACI18991.1	B5YDK3	520	11–458
15	GBE	Bacteria	*Elusimicrobium minutum*	ACC97615.1	B2KAR9	537	12–470
16	GBE	Bacteria	*Fibrobacter succinogenes*	ACX74327.1	UPI0001A81974	545	12–485
17	GBE	Bacteria	*Frankia inefficax*	ADP79978.1	E3ITT1	535	14–472
18	GBE	Bacteria	*Granulicella mallensis*	AEU36022.1	G8NPL0	621	17–509
19	GBE	Bacteria	*Herpetosiphon aurantiacus*	ABX07093.1	A9AZN9	649	12–583
20	GBE	Bacteria	*Kosmotoga olearia*	ACR79103.1	C5CDT4	533	11–473
21	GBE	Bacteria	*Kosmotoga pacifica*	AKI97624.1	A0A0G2ZDK6	531	11–473
22	GBE	Bacteria	*Leptospirillum ferriphilum*	AFS54579.1	J9ZDS7	532	12–468
23	GBE	Bacteria	*Leptotrichia* sp. oral taxon 847	AMD94847.1	A0A0X8JTT9	530	11–469
24	GBE	Bacteria	*Mesotoga prima*	AFK07143.1	I2F5E0	534	11–473
25	GBE	Bacteria	*Methylacidiphilum infernorum*	ACD83633.1	B3DWD0	550	34–493
26	GBE	Bacteria	*Mycobacterium tuberculosis*	ABQ74844.1	A5U744	526	17–470
27	GBE	Bacteria	*Nostoc* sp. PCC 7120	BAB74149.1	Q8YUA2	529	11–470
28	GBE	Bacteria	*Paenibacillus mucilaginosus*	AEI39347.1	F8FR97	996	20–489
29	GBE	Bacteria	*Petrotoga mobilis*	ABX31322.1	A9BH92	538	13–475
30	GBE	Bacteria	*Phycisphaera mikurensis*	BAM04561.1	I0IH23	569	14–501
31	GBE	Bacteria	*Pseudothermotoga hypogea*	AJC73354.1	A0A0X1KQ71	535	11–470
32	GBE	Bacteria	*Pseudothermotoga thermarum*	AEH51865.1	F7YW93	526	10–469
33	GBE	Bacteria	*Pseudothermotoga lettingae*	ABV34332.1	A8F848	527	10–469
34	GBE	Bacteria	*Thermosulfidibacter takaii*	BAT71996.1	A0A0S3QUJ6	529	13–471
35	GBE	Bacteria	*Thermus scotoductus*	ADW20837.1	E8PJY5	527	9–464
36	GBE	Archaea	*Palaeococcus pacificus*	AIF69759.1	A0A075LTR5	652	11–474
37	GBE	Archaea	*Pyrococcus* sp. NCB100	AMM54710.1	A0A127BBJ9	634	10–472
38	GBE	Archaea	*Pyrococcus yayanosii*	AEH24426.1	F8AIV1	564	10–473
39	GBE	Archaea	*Thermococcus barophilus*	ALM76435.1	A0A0S1XFE3	666	11–476
40	GBE	Archaea	*Thermococcus* sp. AM4	EEB74149.1	B7R210	696	10–470
41	AAMY	Archaea	*Methanocaldococcus jannaschii*	AAB99631.1	Q59006	467	8–357
42	AAMY-like	Bacteria	*Bacteroides thetaiotaomicron*	AAO79410.1	Q89ZS1	460	9–351
43	4AGT	Bacteria	*Dictyoglomus thermophilum*	CAA30735.1	P09961	686	12–367
44	4AGT	Archaea	*Archaeaoglobus fulgidus*	ABW95829.1	A9QMB3	659	11–358
45	4AGT	Archaea	*Pyrococcus furiosus*	AAA72035.1	P49067	649	12–359
46	4AGT	Archaea	*Thermococcus kodakarensis*	BAA22062.1	O32450	653	11–358
47	4AGT	Archaea	*Thermococcus litoralis*	BAA22063.1	O32462	659	11–358
48	APU	Bacteria	*Dictyoglomus turgidum*	ACK41960.1	B8DZM7	1042	48–552
49	APU	Bacteria	*Spirochaeta thermophila*	ADN02534.1	E0RN65	1000	39-532
50	APU	Archaea	*Pyrococcus furiosus*	AAB71229.1	O30772	853	39–574
51	APU	Archaea	*Sulfolobus acidocaldarius*	AAY80509.1	Q4J9M2	884	336–725

52	APU	Archaea	*Thermococcus hydrothermalis*	AAD28552.1	Q9Y8I8	1337	40–574
53	APU	Archaea	*Thermococcus kodakaraensis*	BAD85963.1	Q5JJ55	1100	40–575
54	APU	Archaea	*Thermococcus litoralis*	BAC10983.1	Q8NKS8	1089	37–570
55	APU	Archaea	*Thermococcus siculi*	ACJ03924.1	B6SED6	1351	40–574
56	APU-CMD	Archaea	*Caldivirga maquilingensis*	ABW02197.1	A8M8X8	606	114–480
57	APU-CMD	Archaea	*Desulfurococcus amylolyticus*	ACL10679.1	B8D3J8	625	116-496
58	APU-CMD	Archaea	*Staphylothermus marinus*	ABN70497.1	A3DPD7	639	126–508
59	APU-CMD	Archaea	*Thermophilum pendens*	ABL77620.1	A1RWP0	609	113-495
60	AMY	Bacteria	Uncultured bacterium	AEC23345.1	F4ZW71	544	10–434
61	MGA	Archaea	*Pyrococcus furiosus*	AAL80994.1	Q8U2G5	597	10–452
62	MGA	Archaea	*Pyrococcus* sp. ST04	AFK22464.1	I3RE04	597	10–453
63	MGA	Archaea	*Thermococcus cleftensis*	AFL95073.1	I3ZTN9	575	10–436
64	AGAL	Archaea	*Pyrococcus furiosus*	AAG28455.1	Q9HHB5	364	7–339

[a] GBE, glucan branching enzyme; AAMY, α-amylase; AAMY-like protein, α-amylase-like protein; 4AGT, 4-α-glucanotransferase; APU, amylopullulanase; APU-CMD, amylopullulanase/cyclomaltodextrinase; AMY, non-specified amylase; MGA, maltogenic amylase; AGAL, α-galactosidase.
[b] The asterisk signifies the GBEs with already solved tertiary structure.

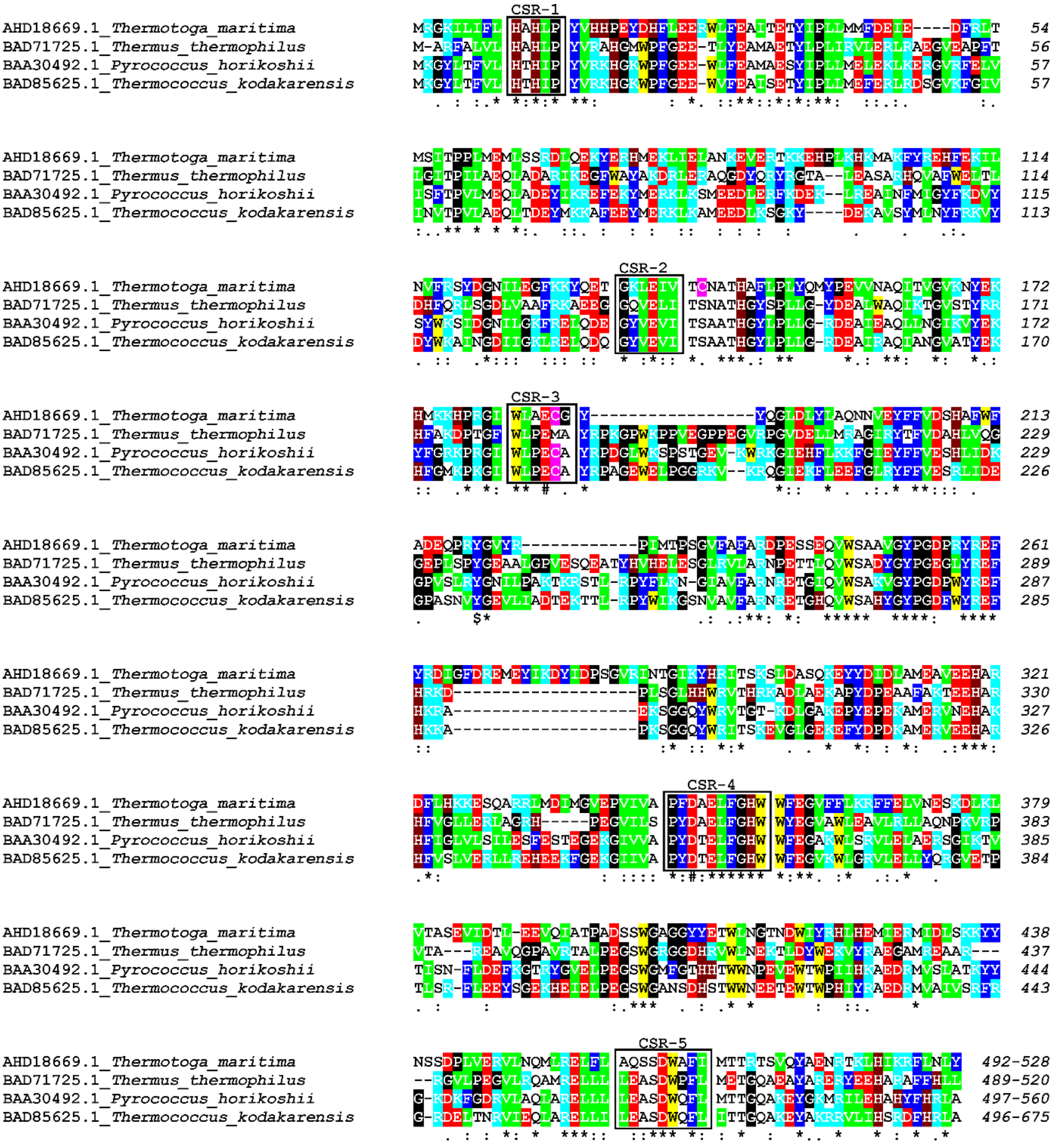

Fig S1. Sequence alignment of the three confirmed GH57 GBEs (*Thermus*, *Pyrococcus* and *Thermococcus*) with AmyC from *Thermotoga maritima*. The alignment spans almost complete sequences from their N-termini and includes the complete family GH57 catalytic domain with the catalytic $(\beta/\alpha)_7$-barrel (containing the first four CSRs) and the essential bundle of a few α-helices (containing CSR-5). Colour code for the selected residues: W - yellow; F, Y - blue; V, L, I - green; D, E - red; R, K - cyan; H - brown; C - magenta; G, P - black. The positions of the two catalytic residues and the tyrosine important for the branching enzyme activity are signified by hashtags and a dollar symbol, respectively, below the alignment. Identical and similar residues are marked by asterisks and

colons or dots, respectively. The three additional regions – “catalytic loop”, “distorted helix” and the “lid 2” are signified by ampersand, caret and addition symbols, respectively, above the alignment blocks.

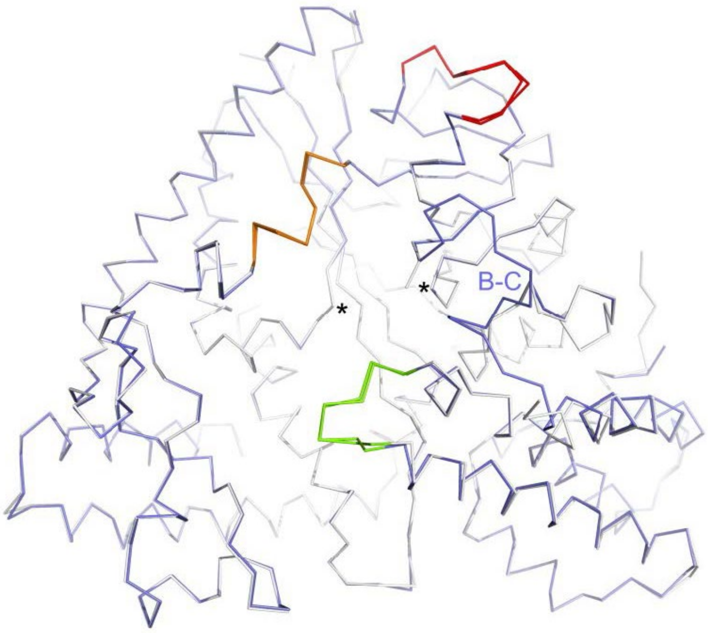

Fig S2. Superposition of the structure of AmyC (light grey) and the homology model of *K. pacifica* putative GBE (blue) showing their highly similar Cα trace, including the catalytic loop (red), distorted helix (orange) and lid 2 (green). The positions of the catalytic residues are indicated with asterisks. The *K. olearia* and *M. prima* GBE homology models GBE (not shown) display a virtually identical Cα trace, except for the loop between domains B and C.
